# Probing Peptide Assembly and Interaction via High-Resolution Imaging Techniques: A Mini Review

**DOI:** 10.3390/ijms26093998

**Published:** 2025-04-23

**Authors:** Xiaoming Zhang, Zhanshu Yang, Jiaxuan Lin, Wei Zhou, Nan Sun, Yi Jia

**Affiliations:** 1 School of Science, Minzu University of China, Beijing 100081, China; 13459868698@163.com (Z.Y.); xsjljx@163.com (J.L.); dreamhunter675@163.com (W.Z.); 2 Optoelectronics Research Centre, Minzu University of China, Beijing 100081, China; 3 National Engineering Research Center for Colloidal Materials, School of Chemistry and Chemical Engineering, Shandong University, Jinan 250100, China; sunnan@sdu.edu.cn; 4 Hubei Key Laboratory of Novel Reactor and Green Chemical Technology, School of Chemical Engineering and Pharmacy, Wuhan Institute of Technology, Wuhan 430205, China; 5 Beijing National Laboratory for Molecular Sciences, CAS Key Laboratory of Colloid, Interface and Chemical Thermodynamics, Institute of Chemistry, Chinese Academy of Sciences, Beijing 100190, China

**Keywords:** peptide molecules, self-assembly, super-resolution microscopy, biomaterials, nanotechnology

## Abstract

Peptide molecules, as fundamental structural units in biological systems, play pivotal roles in diverse biological processes and have garnered substantial attention in biomolecular self-assembly research. Their structural simplicity and high design flexibility make peptides key players in the development of novel biomaterials. High-resolution imaging techniques have provided profound insights into peptide assembly. Recently, the development of cutting-edge technologies, such as super-resolution microscopy (SRM) with unparalleled spatiotemporal resolution, has further advanced peptide assembly research. These advancements enable both the mechanistic exploration of peptide assembly pathways and the rational design of peptide-based functional materials. In this mini review, we systematically examine the structural diversity of peptide assemblies, including micelles, tubes, particles, fibers and hydrogel, as investigated by various high-resolution imaging techniques, with a focus on their assembly characterization and dynamic process. We also summarize the interaction networks of peptide assemblies with proteins, polymers and microbes, providing further insight into the interactions between peptide assemblies and other molecules. Furthermore, we emphasize the transformative role of high-resolution imaging techniques in addressing long-standing challenges in peptide nanotechnology. We anticipate that this review will accelerate the advancement of peptide assembly characterization, thereby fostering the creation of next-generation functional biomaterials.

## 1. Introduction

As essential structural units in biological systems, peptide molecules play a crucial role in life activities. Research on the assembly behavior of peptide molecules and their assemblies can reveal the principles of life activities and deepen our understanding of the mechanisms underlying biological processes [1,2,3,4,5]. Due to the diverse interaction modes between peptide molecules, including van der Waals forces, hydrogen bonding, π-π stacking, electrostatic interactions, and hydrophobic effects, peptide assemblies exhibit rich kinetic and thermodynamic structures [6]. This phenomenon is not only central to biological systems, where it underpins the formation of functional protein complexes and amyloid structures, but also serves as a powerful tool in materials science and nanotechnology based on peptide assembly materials [7,8,9,10]. By altering external conditions, such as temperature, ionic strength, pH, solvent, enzymes, magnetic fields, and electric fields, peptide molecules can assemble into various functional micro- and nanostructures [11], including spherical [12,13], tubular [14], and sheet-like forms [15]. As biomolecules, short peptides possess advantages, such as simple structures, tunable properties, easy chemical modification, high design flexibility, excellent biocompatibility, and biodegradability [16,17]. These characteristics have made them a research hotspot in the field of biomolecular self-assembly and have garnered significant attention in the development of novel biomaterials [10,18,19,20]. In recent years, nanomaterials based on short peptide self-assembly have been extensively studied in the biomedical field, demonstrating immense application potential in multiple domains, such as drug delivery [10], antibacterial applications [15], rapid medical hemostasis [21], medical detection and diagnosis [22], biological 3D printing [23] and tissue engineering [19]. Peptide assemblies have become a central point in the research of novel biomaterials. In-depth research into short peptide assembly materials and their assembly mechanisms holds significant importance for not only the design and development of novel short peptide-based materials but also for both advancing fundamental science and developing innovative technologies.

The investigation of peptide self-assembly relies on a variety of experimental techniques to probe the structural, dynamic, and mechanistic aspects of the process. Traditional characterization methods, such as X-ray crystallography [24], circular dichroism (CD) [25], Fourier-transform infrared spectroscopy (FTIR) [26], and nuclear magnetic resonance (NMR) [27], have provided valuable insights into peptide conformations and interactions. Scanning electron microscopy (SEM) provides detailed surface morphology and three-dimensional images of peptide assemblies, making it ideal for studying surface morphology, hierarchical structures, well-ordered nanotube structures, and interconnected network structures of peptide-based hydrogels [28,29]. However, limitations of these conventional techniques in resolution, sensitivity and the ability to capture dynamic processes in real time or under in situ conditions have limited a comprehensive understanding of the peptide assembly process. They also have limitations on resolving the following questions, for instance, real-time dynamics of nucleation and elongation processes, heterogeneous populations coexisting in equilibrium states, and molecular-level interaction patterns at solvent interfaces. Therefore, cross-scale, highly sensitive in situ characterization techniques demonstrate unique advantages in peptide assembly research [30].

In recent years, high-resolution imaging techniques have revolutionized this paradigm by enabling direct visualization at sub-nanometer resolution. Atomic force microscopy (AFM) and transmission electron microscopy (TEM) have been widely used to visualize the morphology of peptide assemblies, providing critical insights into their structural organization and assembly mechanisms [31,32]. As exemplified by recent breakthroughs, AFM has been a powerful tool for studying peptide assemblies at the nanoscale, offering high-resolution topographical imaging under near-native conditions, as well as providing information on the formation and mechanical properties of peptide nanostructures [33,34]. TEM provides ultra-high-resolution imaging of peptide assemblies, and cryo-electron microscopy (Cryo-TEM) resolves 2–3 Å features in hydrated assemblies, enabling the visualization of internal structures and fine details at the nanoscale level [28,35].

Recently, the super-resolution microscopic imaging (SRM) method that breaks the optical diffraction limit has attracted the attention of scientists in many fields [36,37,38,39,40,41,42]. The 2014 Nobel Prize in Chemistry was awarded to three scientists who have made outstanding contributions to SRM imaging, further recognizing the contribution of SRM imaging technology [43,44]. Generally speaking, SRM can be divided into three classes, including structured illumination microscopy (SIM), stimulated emission depletion microscopy (STED) and single-molecule localization microscopy (SMLM). SIM, a super-resolution method (~100 nm lateral resolution), employs periodic structured light, often sinusoidal patterns, to illuminate samples. The interaction between the structured light and the sample’s fluorescence generates Moiré fringes, encoding high-frequency spatial data. These fringes are captured by a CCD through the optical system, downshifting the high-frequency components to a detectable range. By acquiring multiple images at varying phases and orientations and using computational reconstruction, the high-frequency details are recovered, resulting in a super-resolution image [45]. Its compatibility with live-cell imaging makes it valuable for dynamic process analysis [46]. STED microscopy utilizes two beams: an excitation beam that triggers fluorescence in a diffraction-limited area, and a donut-shaped depletion beam that quenches peripheral fluorophores via stimulated emission. This selective quenching ensures only the center molecules fluoresce, narrowing the point spread function (PSF) and surpassing the diffraction limit to enhance spatial resolution [47]. It excels in imaging dense neuronal synapses and organelle membranes. SMLM encompasses advanced techniques, such as photoactivated localization microscopy (PALM), stochastic optical reconstruction microscopy (STORM), and direct stochastic optical reconstruction microscopy (dSTORM). PALM employs photoswitchable proteins, activating sparse subsets for brief fluorescence before bleaching, repeated cyclically. STORM uses reversible dyes, activating small fractions sequentially. In both methods, the sparse activation strategy prevents overlap of individual PSFs, allowing for nanometer-scale localization precision [48].

**Figure 1:**
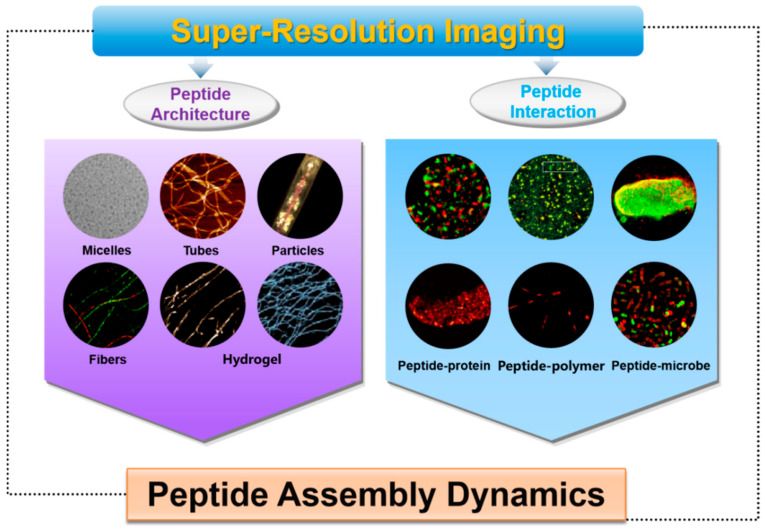
Schematic illustration of super-resolution imaging technology application in peptide assembly dynamics.

Since its advent, SRM has shown remarkable power and application prospects in cell biology, materials science, modern biomedicine and many other fields [49,50]. Thanks to the characteristics of super-spatial and temporal resolution, in situ observation, and multicolor imaging, SRM provides excellent convenience for the study of assembly materials and also becomes a powerful tool for the study of peptide assembly materials [51]. These advanced technologies provide detailed structural information and dynamic insights that are critical for unraveling the molecular mechanisms driving peptide self-assembly [52,53,54]. SRM facilitates the rational design of peptide-based materials by offering direct visualization of assembly pathways, intermediate states, and interactions at the molecular level. This concise review synthesizes key findings on the utilization of SRM in peptide assembly research, placing a strong emphasis on the structural diversity of peptide assemblies—encompassing micelles, tubes, fibrils, and hydrogels—as well as their intricate interactions with proteins, polymers, and microbes (Figure 1). Leveraging these high-resolution imaging techniques, researchers are able to investigate the assembly of peptides and the dynamic interplay among peptide molecules. The integration of SRM into peptide self-assembly research is essential not only for deepening our understanding of this complex process but also for developing next-generation biomaterials with precisely controlled properties and functionalities. This review also helps to underscore the transformative influence of these advancements on the field and their promising potential to surmount significant hurdles in peptide-based nanotechnology.

## 2. Probing Peptide Assembly via High-Resolution Imaging Techniques

While demonstrating remarkable stimulus–response behavior, peptide-based nanostructures capable of forming biomolecular condensates have emerged as crucial molecular architectures with broad applications spanning structural biology to pharmaceutical development. Their dynamic self-organization mechanisms and hierarchical assembly pathways, however, pose significant analytical challenges due to inherent structural polymorphism and transient intermediate states. This complexity necessitates the development of integrated multi-modal imaging platforms that synergistically combine advanced characterization techniques with nanoscale resolution to elucidate the structure–function relationships of these supramolecular assemblies. High-resolution imaging techniques have integrated multiscale characterization techniques and dynamic processes to unravel complex biomolecular interaction networks.

### 2.1. Micelles

Short bioactive peptide sequences are of significant interest in biomaterials development. Peptide amphiphiles (PAs), which self-assemble into dynamic micellar structures capable of multivalent functional peptide display, are being actively engineered for diverse therapeutic and diagnostic applications. Current mechanistic investigations into these dynamic micellar structures leverage advanced analytical methodologies, including small-angle X-ray scattering (SAXS), small-angle neutron scattering (SANS), and cryo-TEM, to elucidate their hierarchical assembly pathways [55].

**Figure 2:**
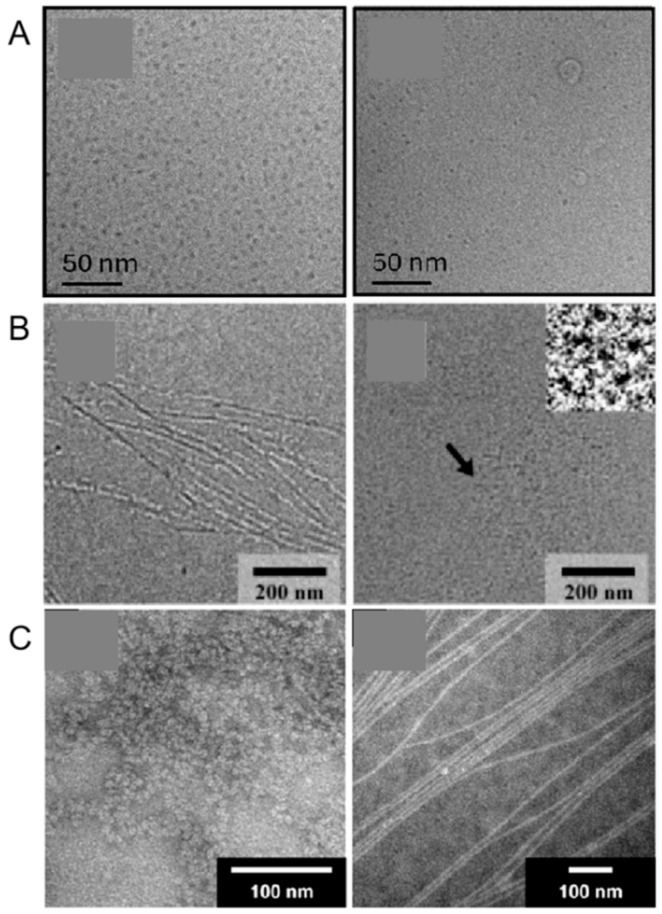
(**A**) Cryo-TEM images from 0.5 wt% aqueous solutions of lipopeptides C_16_-CSK4RGDS and C_16_-CSK4GRDS. Reprinted with permission from Ref. [56]. 2024, American Chemical Society. (**B**) Cryo-TEM images of peptide amphiphile (PA) nanofibers and micelles with an N-methylated glycine near the core. Arrow indicates the location of insets. Reprinted with permission from Ref. [57]. 2006, Paramonov, American Chemical Society. (**C**) The PA micelles formed fibers, and the solution turned into a self-supporting gel after irradiation in the presence of charge-screening calcium chloride salts. Reprinted with permission from Ref. [58]. 2008, European Peptide Society and John Wiley & Sons, Ltd.

Vitreous ice cryo-TEM is the ideal method for investigating the structure of aqueous self-assembling materials because it is free from the large number of artifacts that arise upon drying, such as increased peptide and salt concentration, among others. It has been observed that both the highly cationic CSK4 toll-like receptor agonist hexapeptide sequence, C_16_-CSK4RGDS, which incorporates the RGDS integrin-binding motif, and the control sequence, C_16_-CSK4GRDS, with a scrambled terminal sequence, are capable of forming micelles [56]. Given the significant role of peptide secondary structure in PA self-assembly, modifications to the peptides that either promote or inhibit specific conformations can substantially influence the resulting micellar architecture. C_16_-G7ERGDS PA, designed with N-methylated glycines at various positions, exhibited blocked fiber formation when the modified amino acid was among the four closest to the core due to the absence of hydrogen bonding between neighboring peptides (Figure 2A). Further structural analysis revealed that while hydrogen bonding occurs near the core, the amino acids in the periphery can adopt a weakly ordered conformation (Figure 2B) [57]. When attaching a photocleavable nitrobenzyl group to the N-terminal glycine of C_16_-GAAEERGDS PAs, the PAs initially self-assembled into soluble, spherical micelles with only a small amount of beta-sheet interactions. Upon irradiation, which released the nitrobenzyl group, they transitioned into elongated micelles with strong β-sheet character due to the opportunity for hydrogen bonding at the micelle core (Figure 2C) [58].

However, despite the potential of SRM to provide detailed insights into the self-assembly processes and structural dynamics of peptide amphiphiles, its application in this field has been limited. This is primarily due to several factors: the complexity and cost associated with SRM, the specific sample preparation requirements, and the operational challenges. SRM requires precise control over environmental conditions and often involves intricate sample preparation techniques that may alter the native state of the peptide assemblies. Additionally, the high resolution and sensitivity of SRM can be challenging to achieve consistently, especially for dynamic and delicate structures, such as peptide micelles. As a result, researchers have predominantly relied on more established and accessible techniques, such as SAXS, SANS, and cryo-TEM, for their studies. However, future advancements in SRM technology and sample preparation methods may overcome these limitations, enabling more widespread use of SRM in investigating the intricate structures and functions of peptide amphiphiles.

### 2.2. Tubes

**Figure 3:**
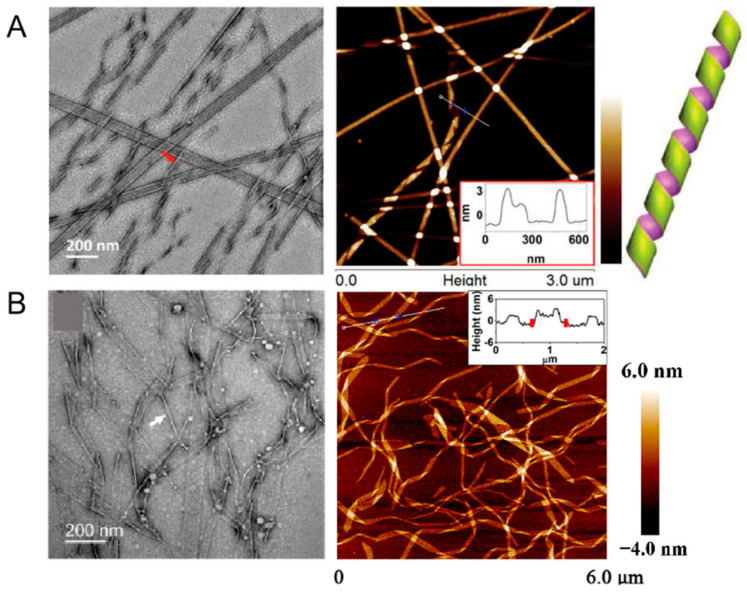
(**A**) Helical intermediates of Ac-KI_3_VK-NH_2_. Red arrow indicates the folded edges of helical ribbons. Bottom right inset is the cross-section profiles of helical ribbons. Reprinted with permission from Ref. [59]. 2018, WILEY-VCH Verlag GmbH & Co. KGaA, Weinheim. (**B**) Self-assembled nanostructures formed by KI_4_K in a mixture of acetonitrile and water with a volume ratio of 20%. The insert box indicates that helical bands, with heights of approximately 2.1 nm and widths mostly ranging from 30−90 nm, are dominant in this sample. Reprinted with permission from Ref. [60]. 2015, American Chemical Society.

Controlling the diameters of nanotubes poses a significant challenge in the self-assembly of nanostructures from templating molecules themselves. To elucidate the underlying mechanism, Zhao et al. designed two series of bolaform hexapeptides, utilizing high-resolution techniques, such as cryo-TEM and negative-staining TEM, to meticulously observe the effects of amino acid substitutions on nanotube dimensions (Figure 3A). This approach enables reliable control over the molecular self-assembling processes, highlighting the importance of molecular design in nanotube formation [59].

Manipulating self-assembly through solvent-controlled structural transitions also holds substantial significance. A designed symmetric amphiphilic peptide, KI_4_K, exhibited morphological transitions among different one-dimensional nanostructures—ranging from wide nanotubes to helical nanoribbons, and subsequently to thin nanofibrils—with increasing acetonitrile ratio. A suite of high-resolution tools, including AFM, CD, FTIR, and SANS, was employed [60]. These sophisticated techniques revealed that the incorporation of acetonitrile into water significantly influenced the hydrophobic interactions among the hydrophobic side chains, while having a minimal impact on the β-sheet hydrogen bonding between the peptide backbones. In addition to the dominant nanotubes formed by KI_4_K in water, nanotubes with helical markings, fragmented nanotubes with clear openings, and short ribbons were also observed from AFM imaging (Figure 3B). The integration of these high-resolution methods provided unprecedented insights into the molecular-level processes governing nanotube formation and structural modulation, demonstrating the power of advanced imaging and spectroscopic technologies in achieving precise control over self-assembled nanostructures.

Unfortunately, we have not found any cases where SRM technology has been used to study the structure and dynamics of peptide nanotubes. The absence of such studies may be attributed to the limitations of fluorescence SRM. Peptide nanotubes, with their delicate structures, may not always withstand the preparation procedures, such as surface fluorescent functionalization or immobilization. In visualizing the fine internal structures, TEM and AFM can provide clearer insights into the morphology and arrangement of nanoscale features. Despite these challenges, we recognize the significant potential of SRM technology in advancing the study of peptide micelles and tubes. SRM instruments and techniques can provide detailed information about the surface properties and interactions, which is crucial for understanding their functional behavior. SRM also allows for real-time observation of nanoscale processes, which could be valuable in studying the self-assembly and stability of peptide nanotubes under various conditions. Future advancements in SRM might expand its applicability to this field.

### 2.3. Particles

Although these static methods ensure higher resolution imaging, they require complicated sample processing and preparation, including fixing and slicing cells, prior to observation. Furthermore, imaging live specimens is virtually impossible with these methods, limiting their use to non-viable objects. As a result, dynamic and real-time information is difficult to obtain using static methods. Since many biomaterials, including peptide nanomaterials, are inherently non-fluorescent, visualizing and tracking their activities both in vitro and in vivo remains challenging. Enhanced dark-field hyperspectral imaging (EDF-HSI), which integrates dark-field microscopy (DFM) and hyperspectral imaging (HSI), is an innovative optical tool with significant potential in bio-related imaging. It allows for the visualization and quantitative measurement of samples in their native biological environments.

**Figure 4:**
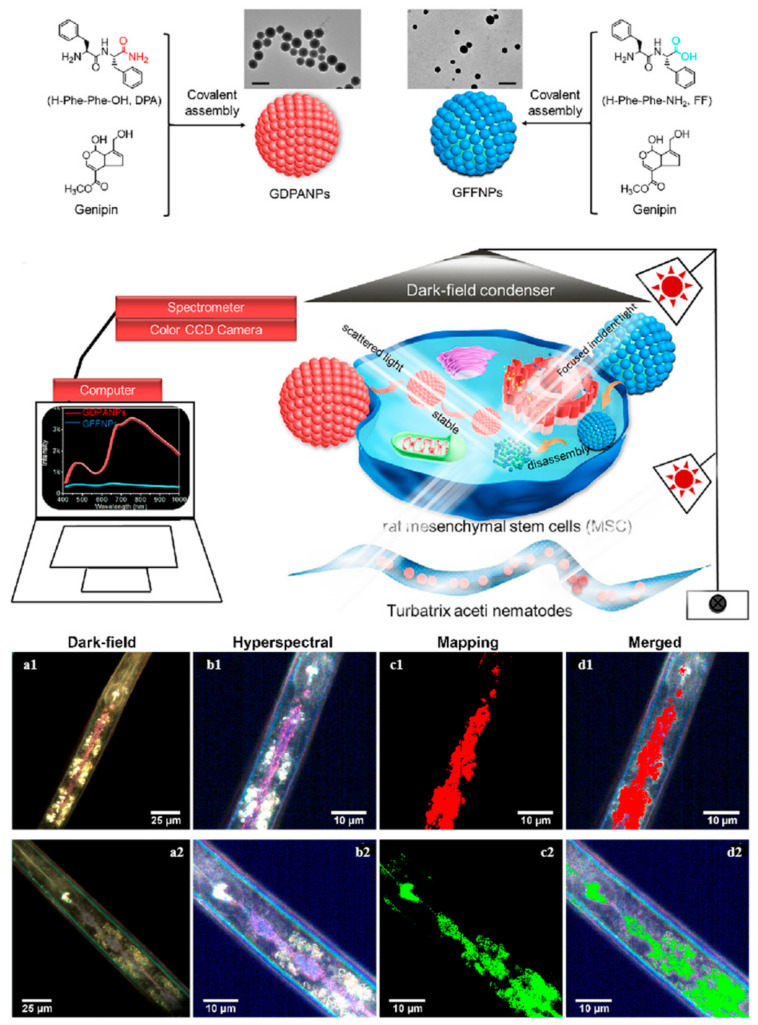
Self-assembled peptide nanoparticles for enhanced dark-field and hyperspectral imaging. (**a1**) Dark-field images in the intestines of *T. aceti* worms; (**b1**) hyperspectral images; (**c1**) corresponding maps; (**d1**) dark-field results of GDPANPs. (**a2**) Dark-field images in the intestines of *T. aceti* worms; (**b2**) hyperspectral images; (**c2**) corresponding maps; (**d2**) dark-field results of GFFNPs. The bright spots in the dark field plot indicate strong scattering signals from the particles. The colors in the hyperspectral plot represent light scattering intensity at different wavelengths. The mapping shows the degree and location of the match with the standard nanoparticle spectrum, using different colors to differentiate material types. Brighter colors indicate a higher degree of match with the standard nanoparticle scattering spectrum. Reprinted with permission from Ref. [61]. 2021, Elsevier B.V.

Yan and his colleagues conducted an investigation into two distinct types of nanoparticles: one derived from diphenylalanine (H-Phe-Phe-OH) and the other from C-terminal amidated diphenylalanine (H-Phe-Phe-NH_2_). The light scattering characteristics of these peptide nanoparticles were found to be predominantly influenced by the constituent peptide building blocks. Furthermore, these building blocks were observed to significantly affect the stability of the resultant nanoparticles when suspended in cell culture media (Figure 4). The zeta potentials of GDPANPs (C-terminally amidated) and GFFNPs (C-terminally carboxylated) are +31.9 mV and −29.2 mV, respectively. GFFNPs carry negative charges from ionized carboxyl groups under physiological conditions, leading to increased electrostatic repulsion, reduced hydrophobicity, and a tendency to disassemble. In contrast, GDPANPs, lacking free C-termini, form more compact and ordered supramolecular structures, enhancing nonlinear light scattering and refraction. Additionally, the positive surface charge of GDPANPs promotes electrostatic interactions with negatively charged cell membranes, facilitating cellular uptake. Therefore, although both nanoparticles scatter most strongly in the near-infrared range (650–800 nm), GDPANPs produce significantly stronger scattering signals and clearer dark-field images. This research marks a pioneering achievement in the visualization and tracking of peptide nanomaterials using EDF-HSI technology. This technology offers opportunities to investigate the interactions of peptide nanomaterials with cells or organisms using noninvasive optical methods. Additionally, given the presence of amino groups in many peptides and proteins, the strategy for synthesizing biomolecule-based nanoparticles is versatile, which will be advantageous for research aimed at visualizing the interactions of non-fluorescent biomaterials with organisms [61].

### 2.4. Fibers

**Figure 5:**
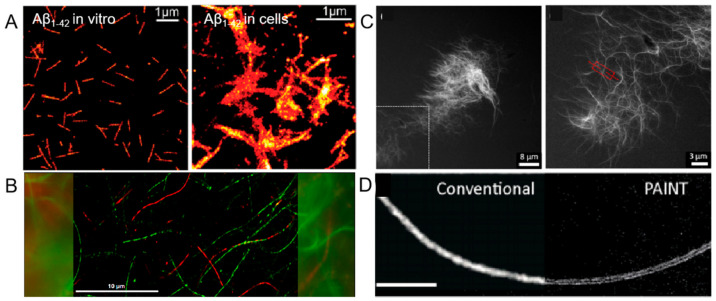
(**A**) dSTORM images of Aβ_1-42_ in vitro and in cells. Reprinted with permission from Ref. [62]. 2011, American Chemical Society. (**B**) Two-color STROM image of I_3_K peptide fibrils. Red represents Alexa Fluor 647 dye-labeled I_3_K peptide fibers, while green represents Cy3B dye-labeled I_3_K peptide fibers. Most fibers are either pure red or pure green, with minimal entanglement or in contact, suggesting the high stability of the I_3_K fibers structure and its resistance to monomer exchange. Reprinted with permission from Ref. [63]. 2017, American Chemical Society. (**C**) STED micrographs of self-assembled short peptide sequence FFALGLAGKK. The image on the right is a magnification of the box. Reprinted with permission from Ref. [66]. 2020, American Chemical Society. (**D**) Comparison of conventional TIRF imaging and PAINT imaging of an FF nanofiber. Scale bar 5 µm. Reprinted with permission from Ref. [67]. 2017, The Royal Society of Chemistry.

The misfolding and aggregation of peptides and proteins are central to the pathology of neurodegenerative disorders, with Alzheimer’s disease (AD) as a prime example. In AD, the β-amyloid peptide (Aβ) forms characteristic fibrillar structures, observable as plaques in affected brains. Understanding the dynamics of these structures is crucial for elucidating disease mechanisms and developing therapeutic strategies. Cryo-TEM has been instrumental in preserving the nanostructures of Aβ in solution, thanks to plunge freezing and low-temperature imaging. However, for in situ studies of aggregation and morphology, dSTORM has emerged as a powerful tool, offering sub-20 nm resolution (Figure 5A) [62]. This technique has enabled the differentiation of oligomeric assemblies and mature fibrils, revealing significant morphological differences between in vitro and in vivo species, suggesting distinct pathological pathways. STORM’s capability to quantify exchange kinetics of self-assembled structures by imaging individual fibrils has been pivotal. When combined with AFM, it allows for the detailed study of the dynamic self-assembly of peptides, such as I_3_K. Multicolor STORM imaging further probes monomer exchange dynamics, providing insights into the structural evolution of peptide fibrils and their network properties under aqueous conditions [63]. PA molecules were shown to migrate between fibers, initially evidenced by FRET kinetics. Analysis of fluorescent marker distribution via STORM imaging combined with spatial autocorrelation revealed a bidirectional exchange mechanism involving the expulsion and reincorporation of monomers or molecular clusters. This exchange exhibited spatial heterogeneity, with variable rates observed between fibers and across different regions of the same fiber [64].

Researchers have harnessed super-resolution fluorescence imaging to reveal the structural and dynamic features of peptide nanofibers with high spatiotemporal resolution [64,65,66,67]. By using dyes such as Cy3 and Cy5, they have quantified dye distribution and supported an exchange mechanism involving monomers (Figure 5B) [63]. To overcome the limitations of STORM, such as complex sample preparation and slow acquisition times, a noncovalent fluorescent labeling method for STED-based super-resolution imaging, was developed. This method allows for the direct, multiscale visualization of static structures and the real-time imaging of dynamic processes, such as enzymatic degradation of nanofibers (Figure 5C) [66]. The versatility of the noncovalent labeling method has been demonstrated with various cationic self-assembling peptides and peptide-functionalized gold nanoparticles. To be noted, two-color STORM experiments were performed to investigate the dynamic process of self-assembly after mixing of two separately labeled samples, and the results revealed the formation of long nanofibers via end-to-end connections of short ones. This approach has also been applied to diphenylalanine (FF)-based nanostructures, using Cy5-FF as a Points Accumulation for Imaging in Nanoscale Topography (PAINT) probe, enabling visualization across a wide range of scales (Figure 5D) [67].

This methodology paves the way for real-time and in situ imaging of supramolecular structures under physiological conditions, fostering the design of synthetic peptides for therapeutic applications and the development of bioresponsive materials. The integrated use of advanced imaging techniques, such as dSTORM, AFM, and STED, along with super-resolution PAINT imaging, has provided a comprehensive understanding of peptide fibril dynamics. These methodologies not only shed light on the molecular mechanisms underlying protein deposition diseases but also facilitate the design of novel biomaterials with potential therapeutic applications.

### 2.5. Hydrogel

The gel-like aggregates observed in neurodegenerative diseases, such as Alzheimer’s, not only form filaments but also present a complex challenge due to the lack of robust quantitative theories. These theories are crucial for linking molecular-scale structure and dynamics of gels to their macroscale performance, thereby facilitating the creation of new high-performance materials through a fundamental bottom-up approach.

**Figure 6:**
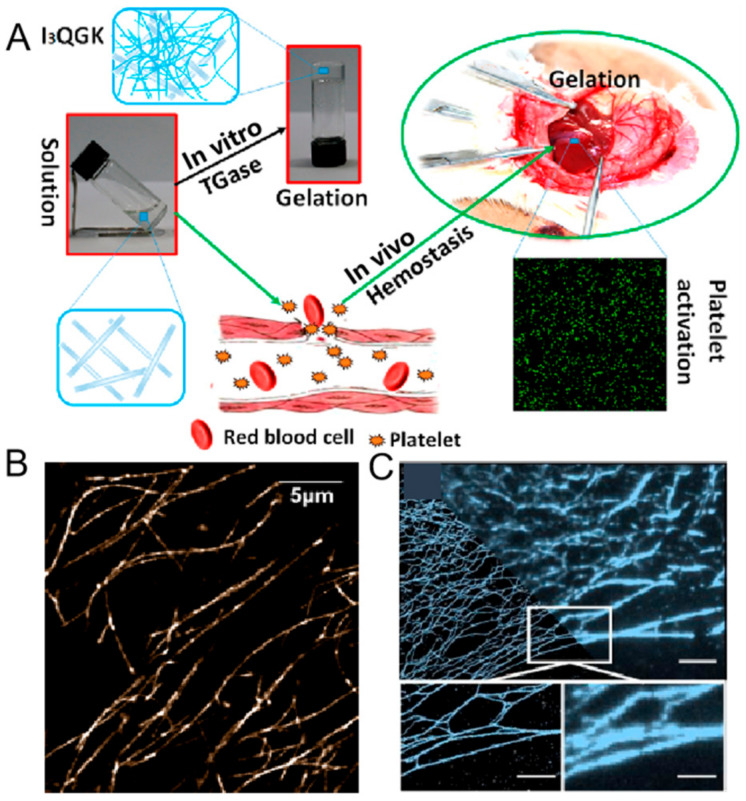
(**A**) Self-assembly schematic of I_3_QGK in clinical hemostasis. Reprinted with permission from Ref. [21]. 2016, American Chemical Society. (**B**) STORM image of the fully labeled I_3_K network, showing regions of densely cross-linked fibrils. Reprinted with permission from Ref. [68]. 2018, American Chemical Society. (**C**) Probe reversibly binds to fibers to form PAINT image (**left**) vs. low-resolution image (**right**). The white box indicates the zoomed-in area displayed in the subfigure. Scale bars: top image, 5 µm; bottom images, 2.5 µm. Reprinted with permission from Ref. [69]. 2020, Chemistry Europe.

To seamlessly integrate peptide self-assembly with enzymatic catalysis, researchers designed an amphiphilic short peptide, I_3_QGK, which self-assembles into long nanoribbons in aqueous solution [21]. Upon introducing transglutaminase, the peptide solution undergoes a distinct sol–gel transition, culminating in the formation of a rigid hydrogel (Figure 6A). This hydrogel exhibits robust shear-thinning and immediate recovery properties, showcasing its dynamic nature. Detailed analyses reveal the presence of substantial nanofibers alongside the original nanoribbons, indicating a complex microstructure. The enzymatic conversion of peptide monomers into dimers through intermolecular ε-(γ-glutamyl) lysine isopeptide bonding drives the rapid self-assembly of these flexible and entangled nanofibers, which, in conjunction with the original nanoribbons, play a pivotal role in the hydrogelation process.

Recent advancements in single-molecule experiments, focusing on gel dynamics, have led to the development of new models. Researchers employed a STORM optical setup to image and describe the dynamics of individual I_3_K peptide fibrils, revealing that single molecules within gels significantly influence various physical phenomena (Figure 6B) [68]. These include the elastic modulus, relaxation times, adhesion, phase separation, wetting behavior, and response to external actuation, such as electric or magnetic fields, and temperature changes. Such insights are invaluable for the future development of designer synthetic peptide-based materials, where specific physical properties are dictated by the mesoscale gel network structure. They identified prestress within the network, showing that the self-prestressed state (SPS) is influenced by peptide concentration and pH, with fiber energy exhibiting a Lévy distribution. Dynamic Fourier decomposition separated thermal fluctuations from SPS-induced compression, yielding a persistence length of 2.41 ± 0.07 mm. A mixture model combining normal and Lévy distributions distinguished unstressed fibers from prestressed ones, quantifying the proportion of stressed fibers at different concentrations.

Furthermore, a single molecule localization microscopy (SMLM) method, based on PAINT, has been developed for the imaging of FmocFF hydrogels in their native state and without direct gel labeling, achieving enhanced resolution (≈50 nm) in both 2D and 3D (Figure 6C) [69]. It enables the extraction of quantitative data, including mesh size and fiber diameter, thereby serving as a valuable complement to existing hydrogel characterization tools and providing crucial information for the design of new materials.

## 3. Probing Peptide Interaction via High-Resolution Imaging Techniques

### 3.1. Peptide–Protein Interaction

**Figure 7:**
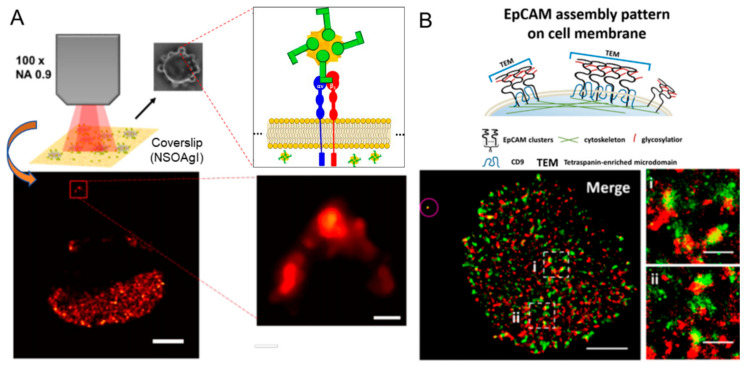
(**A**) Super-resolution SERS imaging of AuNS-RGDFC in cancer cells. RGDFC functionalized AuNS were used as label-free SERS probes and super-resolution SERS results for fixed cells incubated with AuNS-RGDFC recorded at 10 Hz. The scale bar is 2 μm for the main image and 150 nm for the enlarged view. Reprinted with permission from Ref. [70]. 2020, American Chemical Society; (**B**) Schematic illustration of EpCAM assembly pattern on cell membrane. Dual-color dSTORM images revealing the spatial relationship between EpCAM (green) and CD9 (red) on MCF-7 cell membranes, with magnified views of two regions (i) and (ii) showing the co-localization of CD9 and EpCAM. The microsphere, circled in the upper left corner, was used to correct x-y drift and optical registration. Scale bars: 5 μm in main image, and 1 μm in (i−ii). Reprinted with permission from Ref. [71]. 2019, American Chemical Society.

Understanding protein function and advancing molecular biology rely heavily on the ability to locate and identify molecular interactions within cells. Functionalized metallic nanoparticles, such as gold nanostars (AuNS), serve as versatile probes for protein tracking and drug delivery [70]. Their capacity to carry therapeutic agents and their readily functionalizable surfaces make them ideal for these applications. Super-Resolution Surface-Enhanced Raman Scattering (SERS) technique has been employed for imaging and tracking membrane receptors, specifically αvβ3 integrin receptors in colon cancer cells. Peptide-functionalized AuNS (conjugated with RGDFC) target these receptors, producing a bright and fluctuating SERS signal (Figure 7A). The SERS spectrum confirms the interaction between the RGDFC peptide and the integrin receptor. Specific and non-specific binding events are distinguished, with specific binding monitored at a localization precision of approximately 6 nm. Signal fluctuations were attributed to molecular diffusion within the SERS hotspot or reconfiguration of the surface atomic structure. The study revealed that the diffusion coefficient of functionalized AuNS-RGDFC was significantly lower than that of non-functionalized AuNS, indicating strong membrane receptor binding. This diffusion constraint reflects a receptor-anchoring effect, offering a quantitative parameter for probing membrane protein dynamics. Additionally, the branched morphology of gold nanostars renders their SERS signals sensitive to the polarization direction of the electric field. Upon receptor binding, the orientation of AuNS-RGDFC may shift in response to receptor conformation or membrane curvature, leading to a crescent-shaped SERS signal distribution at specific binding sites.

Dual-color dSTORM technology was utilized to reveal the spatial relationship between EpCAM and CD9 on the MCF-7 cell membrane [71]. Additionally, the quality of super-resolution images was assessed by the block Fourier ring correlation (FRC) resolution mapping technique. A comparison between Cy3-conjugated small molecule peptides and antibodies for EpCAM recognition shows enhanced specificity and clustering identification capability of small molecule peptides (Figure 7B). Due to their small size and single binding site, small-molecule peptides are superior to antibodies in super-resolution imaging. They emerge as optimal probe substitutes, offering higher specificity and better ability to identify protein clusters. Using dSTORM and fluorophore-labeled peptides targeting EpCAM, partial co-localization of EpCAM with the tetraspanin CD9 was observed. CD9 knockdown resulted in a reduction and dispersal of EpCAM clusters. dSTORM analysis further revealed that EpCAM organization on the membrane is constrained by both the cytoskeleton and glycosylation, leading to heterogeneous cluster formation with variable sizes.

**Figure 8:**
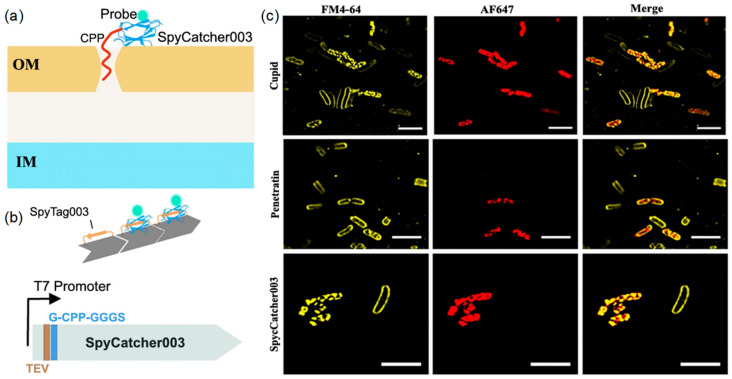
(**a**) Diagram of hypothesized function of CPP-SpyCatcher fusion proteins; (**b**) Design of the CPP-SpyCatcher fusion; (**c**) Lattice SIM imaging of stained bacteria shows internalization of SpyCatcher and CPP fusions. The overlap of signals from FM4-64 dye-labeled cell membranes (red) and AF647-labeled SpyCatcher003 (yellow) indicates that the probe was successfully transduced into the cytoplasmic lysate of freeze-thawed *E. coli*, even in the absence of CPP sequences. Scale bars: 5 μm. Reprinted with permission from Ref. [72]. 2023, IOP Publishing Ltd.

Three cell-penetrating peptides—CUPID, Pentratin, and pVEC—are used to create fusion protein probes for super-resolution microscopy. This innovative approach aims to bypass the need for prior permeabilization treatments, which can otherwise introduce imaging artifacts [72]. By employing fluorescence and super-resolution Lattice Structured Illumination Microscopy (Lattice SIM), these fusion proteins emerge as versatile tools for labeling proteins or delivering cargo attached to SpyCatcher003 via SpyTag003 across diverse cell types (Figure 8). The studies establish a method for irreversible labeling of target proteins and biomolecules without reliance on plasmid expression. SIM imaging revealed that AF647-labeled fusion proteins exhibited a more homogeneous intracellular distribution compared to unfused SpyCatcher. Protein uptake varied across bacterial species. In *E. coli*, freeze–thaw treatment disrupted the membrane, facilitating probe entry, whereas mid-log phase cells showed negligible uptake. In *B. crescentus*, the CPP sequences markedly enhanced SpyCatcher internalization, reflecting improved uptake efficiency in cells enriched with anionic lipids.

Similarly, a small, PDZ-based, peptide–protein interaction pair was engineered that is genetically encodable and compatible with super-resolution imaging upon cellular fixation without additional labeling [73]. Stoichiometric labeling control by genetic incorporation of this probe into the cellular vimentin network and mitochondria resulted in super-resolved 3D reconstructions with high specificity and spatial resolution. This peptide–protein interaction is compatible with quantitative PAINT, and its binding kinetics remains unaltered upon fixation. This breakthrough enables the use of a broader array of inorganic probes, expanding the possibilities in molecular imaging. The integrated approach of peptide self-assembly, enzymatic catalysis, and high-resolution imaging has vast potential applications in life sciences and biomedicine.

### 3.2. Peptide–Polymer Interaction

Self-assembling peptides (SAPs) are extensively employed as scaffolds in their own right, and more recently, as fillers for microporous scaffolds. In this dual role, SAPs not only provide a cell-friendly nanoenvironment but also enhance the mechanical properties of the scaffolds. The characterization of the interaction between these short peptides and the scaffold material is pivotal in evaluating the potential of such a combined system. In a recent research, the interaction between poly (ethyl acrylate) and an ethyl acrylate–acrylic acid copolymer with the SAP RAD16-I was investigated utilizing AFM. It was demonstrated that the interaction and self-assembly of the peptide are highly sensitive to the wettability and electronegativity of the polymeric substrate [74].

**Figure 9:**
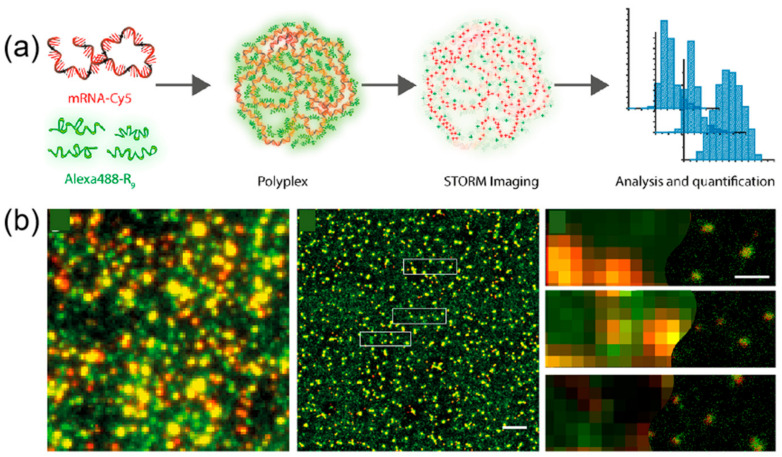
(**a**) Schematic representation of polyplex formation from mRNA-Cy5 and AlexaFluor488-R9; (**b**) Conventional fluorescent image (red represents mRNA molecules and green R9 molecules) and dSTORM image of polyplexes. The yellow spots indicate co-localization of mRNA polyplexes with the R9 peptide. The boxed area in the middle image is magnified in the right panel. Scale bar: 2 μm (middle), 400 nm (right). Reprinted with permission from Ref. [75]. 2019, American Chemical Society.

In the realm of peptide–material interactions, super-resolution imaging techniques, such as dual-color dSTORM, provide nanoscale visualization superior to AFM, crucial for deciphering the molecular architecture of these interactions. dSTORM’s ability to quantitatively analyze individual complexes sheds light on their size, composition, and destabilization in biological settings, facilitating rational design and optimization. Cationic short peptides, such as cell-penetrating peptides (CPPs), play a pivotal role in cellular delivery of genetic material, and their synergy with SAPs can significantly boost the efficiency of therapeutic agent delivery. However, the nanoscale structure of polyplexes has remained largely mysterious due to the challenges in achieving nanometer-resolution visualization of their molecular components. This gap in understanding has been a major obstacle in the direct structural design of polyplexes. Super-resolution imaging emerges as a powerful tool to probe the structure and molecular composition of individual CPP-mRNA polyplexes with unprecedented precision, enabling real-time visualization of CPP-mediated delivery processes and efficiency assessment (Figure 9) [75]. Moreover, quantitative dSTORM of polyplexes complements existing characterization methods, deepening our insight into the factors governing polyplex activity both in vitro and in cellular contexts. Using dSTORM imaging, researchers visualized self-assembled R9-mRNA polymers and identified an optimal N/P ratio of 5 for stable particle formation. dSTORM’s enhanced resolution revealed pronounced colocalization of peptide and mRNA signals, indicating uniform mRNA encapsulation within particles at N/P 5, reflecting maximal structural stability.

**Figure 10:**
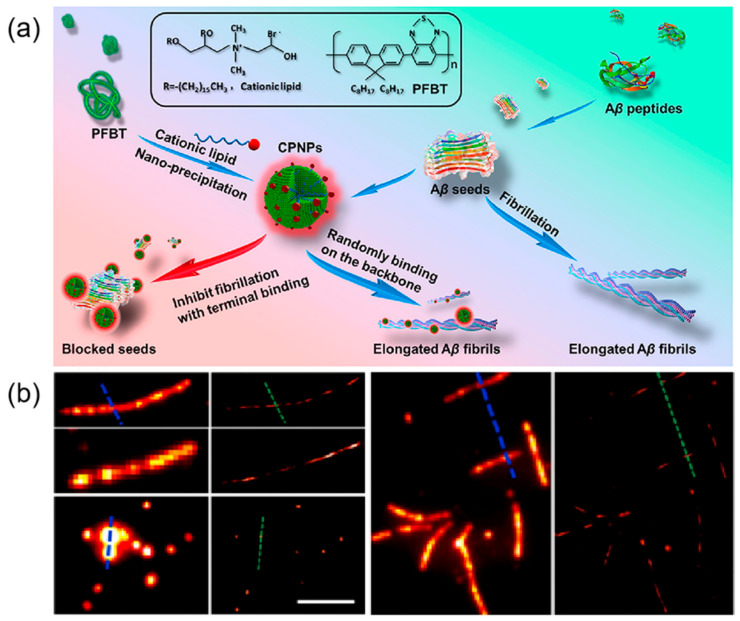
(**a**) Schematic diagram of procedures for CPNPs fabrication and Aβ_1−40_ fibril inhibition with CPNPs; (**b**) Representative conventional fluorescence microscopy images of Aβ_1−40_ fiber filaments and corresponding reconstructed super-resolution images. Scale bar 5 μm. Reprinted with permission from Ref. [76]. 2019, American Chemical Society.

The development of potent inhibitors is crucial for preventing Aβ assembly, a key strategy in the fight against neurodegenerative diseases. High-resolution visualization of these aggregates and their structures is indispensable for advancing biological research. Recent innovative research has led to the creation of conjugated fluorescent polymer nanoparticles (CPNPs), designed to inhibit the fibrillation of Aβ_1–40_ peptides (Figure 10) [76]. These sophisticated nanoparticles are not only ideal for super-resolution imaging but also effectively halt fibrillation by attaching to the ends of seed fibrils, demonstrating their extensive potential in various imaging techniques. CPNPs20, with a zeta potential of +36 mV, adsorb onto the negatively charged termini and backbone of Aβ fibers, with their hydrophobic tail inserting into the Aβ hydrophobic core, stabilizing the structure and inhibiting fibril elongation. Without CPNPs, fiber length increased to ~3.5 ± 0.7 μm at 6 h, while in their presence, fibers measured ~0.4 ± 0.1 μm. CPNPs20’s burst-like scintillation makes them suitable for dSTORM imaging, which achieves ~20 nm resolution compared to ~400 nm for conventional fluorescence microscopy. Imaging showed over 80% of CPNPs20 bound to fiber ends, with additional binding along the backbone. The fusion of self-assembling peptides with cutting-edge imaging technologies presents a holistic approach to enhancing tissue engineering scaffolds, optimizing genetic material delivery, and devising therapeutic strategies for neurodegenerative diseases. This interdisciplinary methodology not only enhances our molecular understanding of peptide interactions but also paves the way for innovative biomedical advancements.

### 3.3. Peptide–Microbe Interaction

**Figure 11:**
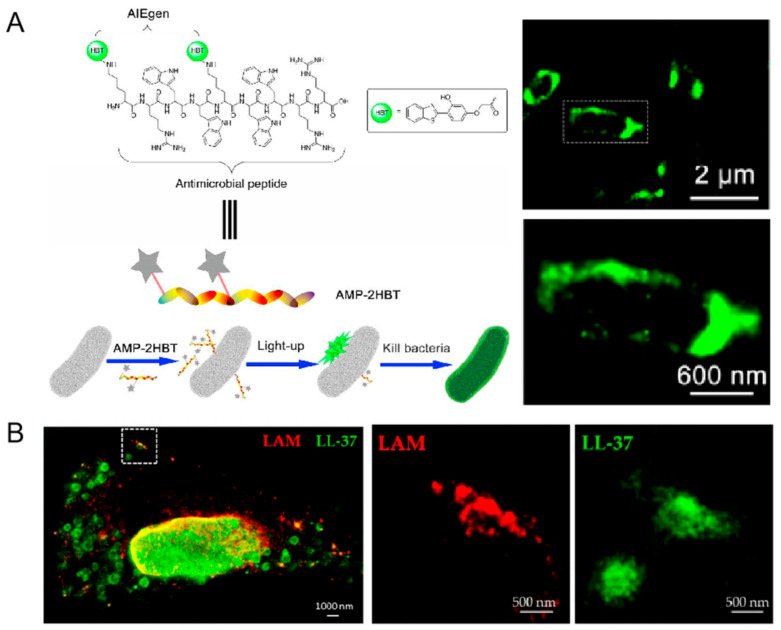
(**A**) AMP-2HBT for bacterial imaging and killing. The white box indicates the area enlarged in the subgraph below. Reprinted with permission from Ref. [77]. 2018, American Chemical Society. (**B**) Demonstration of the antimicrobial effect of LL-37-TAMRA on intracellular Mycobacterium tuberculosis using two-color STED microscopy. The white box indicates the area enlarged in the subgraph with LAM and LL-37, respectively. Reprinted with permission from Ref. [78]. 2020, MDPI.

The escalating threat of multidrug-resistant bacterial infections demands innovative solutions, with antimicrobial peptides (AMPs) emerging as potent alternatives to conventional antibiotics. Despite their promising bactericidal properties, the precise mechanisms of AMP action remain elusive. Fluorescence imaging, renowned for its dynamic monitoring capabilities, user-friendliness, and high sensitivity, has been pivotal in unraveling these mechanisms. A groundbreaking aggregation-induced emission (AIE) active probe, crafted by linking the antimicrobial peptide HHC36 with the AIE fluorogen 2-(2-hydroxybenzothiazol-6-yl)ethanol, has demonstrated exceptional potential in visualizing AMP activity and devising novel strategies against multidrug-resistant bacterial infections (Figure 11A) [77]. By combining the flow-through assay with STORM imaging, it was observed that the assay’s positive rate increased over time. Initial fluorescence signals appeared at 15 min, followed by membrane cluster formation at 30 min, and saturation at 60 min, at which point STORM revealed maximum fluorescence density. Additionally, MP-2HBT exhibited potent bactericidal activity against *E. coli*, achieving a kill rate of 94.33% at 20 μM and 99.53% at 50 μM.

Recent advancements in super-resolution microscopy, particularly STED microscopy, have illuminated the intricate interactions between peptides and pathogens within human macrophages [78]. This study delved into the uptake, intracellular trafficking, and interactions of the antimicrobial peptide LL-37 with both macrophages and the virulent Mycobacterium tuberculosis (Mtb). The findings revealed that LL-37 was internalized by both uninfected and Mtb-infected macrophages, localizing to the membranes of early endosomes and lysosomes—key compartments harboring mycobacteria (Figure 11B). Functionally, LL-37 was observed to disrupt the cell walls of both intracellular and extracellular Mtb, leading to pathogen destruction. STED microscopy, as an innovative and highly informative tool, significantly augments the capabilities of conventional confocal microscopy in studying host-pathogen-peptide interactions.

**Figure 12:**
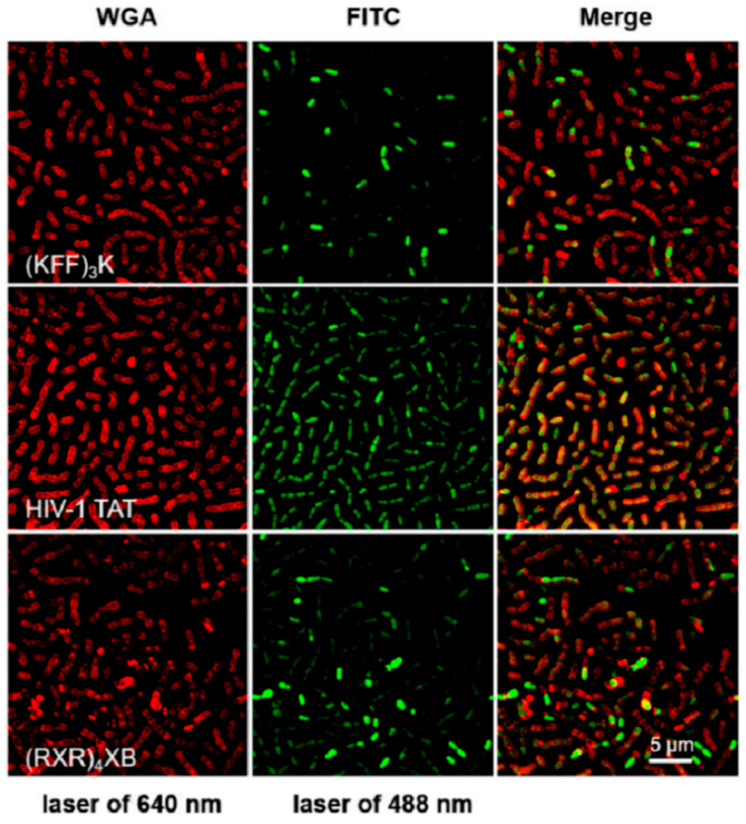
Representative images were used for analyzing the internalization efficiency of FITC-labeled CPPs in *S. suis* by SR-SIM. Reprinted with permission from Ref. [79]. 2024, MDPI.

Cell-penetrating peptides (CPPs), promising vectors for the efficient delivery of antisense oligonucleotides, such as peptide nucleic acids (PNAs), have been explored for their potential to deliver therapeutic agents into bacterial cells, offering a robust strategy against multidrug-resistant bacterial infections [79]. To evaluate the efficacy of four different CPPs for PNA delivery into *S. suis* cells, a comprehensive approach combining super-resolution structured illumination microscopy (SR-SIM), flow cytometry, and toxicity assays was employed. The results highlighted that HIV-1 TAT-conjugated PNA, specifically targeting the essential gyrase A subunit gene (TAT-anti-gyrA PNA), effectively inhibited the growth of *S. suis* (Figure 12). This integrated methodology, merging advanced imaging techniques with peptide-based therapies, not only deepens our understanding of the molecular dynamics of bacterial infections but also paves the way for the development of effective treatments against multidrug-resistant bacteria.

## 4. Perspectives

SRM has allowed the comprehensive understanding of peptide assembly and its processes, observing the dynamic processes of peptide assembly in real time, mapping the spatial distribution and interactions of peptides within assemblies, characterizing its heterogeneous structures and enabling a more comprehensive understanding of the assembly landscape. Despite its transformative potential, the research of peptide self-assembly with the assistance of SRM also faces several challenges.

Firstly, fluorescent labeling is indispensable in SRM imaging. However, the need for fluorescent labeling might sometimes perturb the native behavior of peptides. Developing minimally invasive labeling methods or label-free SRM techniques is a key point for future research.

Secondly, the large datasets generated by SRM imaging require advanced computational tools for analysis and interpretation. With the development of cutting-edge technology, machine learning and artificial intelligence will play a significant role in extracting meaningful insights from complex imaging data [80], helping analyze the research results of peptide self-assemblies.

Thirdly, a single characterization tool can provide important information in peptide assembly studies; however, a single characterization tool also has certain limitations, making it difficult to fully understand the mechanism and properties of peptide assembly. Through the combination of SRM with other multiple technologies and emerging characterization methods, such as AFM, cryo-EM, or spectroscopy, researchers can overcome these limitations and obtain a more comprehensive and accurate understanding of peptide assembly. In the future, interdisciplinary collaboration and technological innovation will continue to drive the development of peptide assembly research, providing a solid foundation for the design and application of novel functional materials.

Finally, with the development of biomedical applications of peptide assemblies, extending SRM imaging to in vivo systems will be a new technical requirement, enabling the study of peptide assemblies in the native biological contexts, opening new possibilities for the deepening of the related biomedical research and applications [81].

Therefore, understanding the fundamental principles that dominate peptide self-assembly not only provides insights into the structural and functional properties of these materials but also enables the rational design of peptide structures and assemblies with tailored properties for specific applications. By elucidating the interactions and pathways involved in peptide assembly, researchers can optimize the formation of nanostructures with desired characteristics, such as enhanced stability, biocompatibility, and functionality. This is crucial for advancing the field of peptide-based materials, paving the way for innovative solutions in biomedicine, nanotechnology, and beyond. Ultimately, a deeper understanding of peptide assembly mechanisms with multitudinous advanced technology tools will drive the creation of next-generation materials with unprecedented precision and performance.
